# A Computational Study of Structure and Reactivity of *N*-Substitued-4-Piperidones Curcumin Analogues and Their Radical Anions

**DOI:** 10.3390/molecules21121658

**Published:** 2016-12-02

**Authors:** Maximiliano Martínez-Cifuentes, Boris Weiss-López, Ramiro Araya-Maturana

**Affiliations:** 1 Programa Institucional de Fomento a la Investigación, Desarrollo e Innovación, Universidad Tecnológica Metropolitana, Ignacio Valdivieso 2409, Casilla 9845, Santiago 8940577, Chile; 2 Departamento de Química, Facultad de Ciencias, Universidad de Chile, Las Palmeras 3425, Casilla 653, Santiago 7800003, Chile; bweiss@uchile.cl; 3 Instituto de Química de Recursos Naturales, Universidad de Talca, Av. Lircay s/n, Casilla 747, Talca 3460000, Chile

**Keywords:** curcumin, 4-piperidone, computational chemistry, radical anion, reactivity indices

## Abstract

In this work, a computational study of a series of *N*-substitued-4-piperidones curcumin analogues is presented. The molecular structure of the neutral molecules and their radical anions, as well as their reactivity, are investigated. *N*-substituents include methyl and benzyl groups, while substituents on the aromatic rings cover electron-donor and electron-acceptor groups. Substitutions at the nitrogen atom do not significantly affect the geometry and frontier molecular orbitals (FMO) energies of these molecules. On the other hand, substituents on the aromatic rings modify the distribution of FMO. In addition, they influence the capability of these molecules to attach an additional electron, which was studied through adiabatic (AEA) and vertical electron affinities (VEA), as well as vertical detachment energy (VDE). To study electrophilic properties of these structures, local reactivity indices, such as Fukui (*f*^+^) and Parr (*P*^+^) functions, were calculated, and show the influence of the aromatic rings substituents on the reactivity of α,β-unsaturated ketones towards nucleophilic attack. This study has potential implications for the design of curcumin analogues based on a 4-piperidone core with desired reactivity.

## 1. Introduction

Curcumin is a natural polyphenol, isolated from the herb *Curcuma longa* L. It has been used in Indian ayurvedic, Chinese and Arabian medicine from ancient times [1,2]. Nowadays, curcumin is used in the food industry as a coloring and flavoring agent. A currently active field of research deals with the multiple biological activities displayed by curcumin and their derivatives [1,3,4,5,6]. Nevertheless, for these applications, curcumin presents some disadvantages, namely poor water solubility, poor bioavailability, and fast metabolic degradation [7,8,9]. 

**Figure 1:**
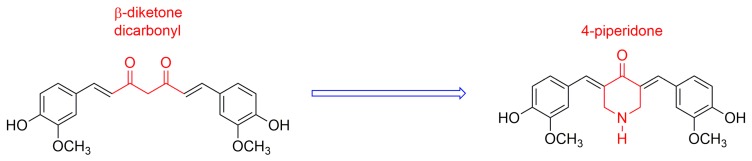
4-piperidone structure derived from curcumin.

In this context, the search for curcumin derivatives and analogues that overcome the above-mentioned disadvantages has become an active area of research [1,2]. The synthesis of new curcumin derivatives has been guided by several approaches to increase the bioavailability of these compounds without losing their pharmacological action [1,2]. The β-diketone moiety, present in curcumin, is a specific substrate for liver enzymes, which contributes to their fast metabolism [10]. Additionally, this feature has been also associated to the generation of reactive oxygen species (ROS), which are involved in several mechanisms of cellular apoptosis [11,12]. Among other possibilities, the replacement of β-diketone moiety by a carbonyl group conjugated by the two sides (Figure 1) has emerged as a new promising alternative [13,14].

Among these compounds, those whose central linker is a 4-piperidone scaffold have shown a high potential as anti-inflammatory and anticancer agents [13,15]. These kinds of compounds have a preferential affinity for thiols, in contrast to amino or hydroxyl groups [16]. Apparently, the central heterocyclic ring fits a primary binding site, being this interaction influenced by the nature of the substituents at the nitrogen atom [17]. The presence of 4-piperidone alkylated at the nitrogen atom, along with phenyl rings bearing a great variety of substituents, constitutes the key features of a series of monocarbonyl analogues of curcumin, whose anti-inflammatory and anti-cancer activities have been recently reported [14,15]. Moreover, dimers linked by the nitrogen atoms, have also shown potent cytotoxicity against cancer cells [18]. 

As far as we know, despite the relevant articles that deal with the pharmacological properties of curcumin derivatives having the 4-piperidone moiety mentioned above, no theoretical reports about the electronic structure and reactivity of this kind of compounds has been published. Based on the above, in this work we carry out a density functional theory (DFT) study of two series of 4-piperidone curcumin (4-PC) analogues, with methyl or benzyl group at the nitrogen atom (Table 1). To gain insight about the chemical reactivity and the determinants of the biological activity of these compounds, both neutral and radical anions are studied. In particular, the effect of aromatic ring substituents on the geometrical and molecular orbital properties, as well as their capability to attach an extra electron, are investigated.

## 2. Results and Discussion

### 2.1. Geometry and Electronic Structure Analysis of Neutral Species

The series of 4-PC derivatives were chosen with the aim of studying the influence of substituents in the aromatic rings and at the nitrogen atom on reactivity. The neutral closed shell molecules were studied at the B3LYP/6-31G(d) level. A conformational analysis at the PM6 level was carried out on the rotation around N_11_-C_12_ and C_12_-C_Ar1_ bonds of compound **7** (Figure S1, Supplementary Materials), and the minimum energy geometry found was used as a model input for the benzyl series. Plots of all optimized structures are presented in the supporting information section. The main minimum energy geometrical parameters are presented in Table S1 (Supplementary Materials).

Compounds **1**–**6** did not show significant structural differences arising from variations in the substituents on the aromatic rings, except for a slight shortening of the C_2_-C_3_ bond and an elongation of the C_3_-C_4_ bond, for **4**, **5**, and **6** when compared with **1**, **2**, and **3**. The replacement of a benzyl by a methyl group as a substituent at the nitrogen atom did not modify geometrical parameters of compounds **7**–**12**, except for the elongation of the N_11_-C_12_ bond. These results show that substitution at the nitrogen atom by a methyl or benzyl groups do not significantly affect the molecular geometry. 

**Figure 2:**
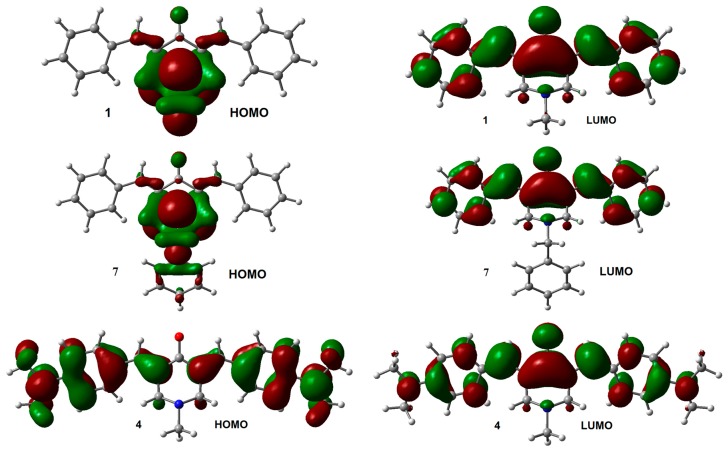

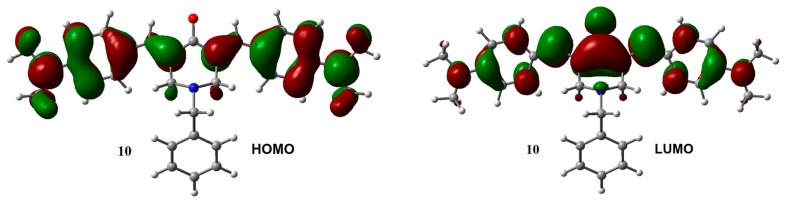
HOMO and LUMO orbitals for neutral species of **1**, **4**, **7**, and **10**. Isosurface value = 0.02 e/Å^3^.

The influence of the substituent on the electronic structure of these molecules was studied by the analysis of their frontier molecular orbitals (FMO), observing differences in FMO distribution patterns for different type of substituents. A similar behavior was observed for the HOMO and LUMO distribution among the members of this series with *N*-methyl (**1**–**6**) and *N*-benzyl (**7**–**12**) substituents. For both unsubstituted aromatic rings molecules (**1** and **7**), the HOMO was fully localized on the 4-piperidone ring and the *exo* double bonds, while the LUMO was distributed through most part of the molecules, except over the substituents at the nitrogen atom. Contrary, the halogen substituted aromatic rings (compounds **2**, **3**, **8**, and **9**), contributed to the HOMO (Figure S2, Supplementary Materials). Instead, the dimethyl-amine derivatives displayed the HOMO localized on the *exo* double bonds as well as over the aromatic rings (compounds **4** and **10**, Figure 2). Electro withdrawing substituents –CN and –CF_3_ (compounds **5**, **6**, **11**, and **12**) distributed their HOMO in a similar way that unsubstituted **1** and **7** do. There are no significant effects from the substituents on the LUMO distribution, for all molecules.

The energies of HOMO and LUMO for the *N*-methyl derivatives **1**–**6** were lower than those for the *N*-benzyl derivatives **7**–**12** (Table 2), except for the 4-dimethylamino derivatives (compounds **4** and **10**) where they were the same for both HOMO orbitals (−4.87 eV), and almost the same for the LUMO orbitals (−1.51 eV and −1.50 eV for **4** and **10** respectively). The HOMO-LUMO gap (GAP_H-L_) was higher for **1**–**6** compared with their analogs **7**–**12** (Table 2), except for **4** whose gap was lower than the one obtained for **10**. The effect of halogens on the energy level of FMO was very similar; decreasing both, HOMO and LUMO energies, like the unsubstituted **1** and **7** derivatives. On the other hand, the effect of dimethylamine group increased the energy levels of the FMO, being more remarkably for HOMO than for LUMO, which leads to a smaller GAP_H-L_ respect to the other analogs. 

Global reactivity indices, such as electronic chemical potential (μ), hardness (η), and electrophilicity (ώ) were also obtained for all molecules according with their definitions (Table 2) [19,20]. η indicates the resistance of the molecule to change their electron density distribution and it was very similar for all compounds, except for **4** and **10**, where the presence of dimethyl-amine substituents decreased η significantly. On the other hand, μ indicates the change in free energy when electrons are added or removed from the molecule, and ώ is a measurement of the trend of a molecule to act as an electrophile, and is obtained from μ and η. Values of ώ increased for **2**, **3**, **5**, and **6** compared with **1**, while it was reduced when compared with **4**. A similar behavior was observed for the benzyl analogues **7** to **12**, being both series very similar respect to their values of ώ. These results show that the tendency to increase ώ is CN > CF_3_ > Cl = Br > N(CH_3_)_2_, in agreement with basic concepts of organic chemistry. 

### 2.2. Radical Anions and Electron Affinities

All molecular orbitals and properties of anion radical species were calculated at the B3LYP/6-31G(d) level. AEAs, VEAs, and VDEs were also calculated at the B3LYP/6-311+G(2df,p)//B3LYP/6-31G(d) level.

Among the main mechanisms invoked to explain the biological activities of this kind of molecules are those involving free radicals [21,22,23]. It has been previously shown that the reduction potentials of this kind of molecules, which present a α,β-unsaturated ketone moiety, can affect directly their biological activity [24,25]. In order to study the anion radical species derived from these curcuminoids, an orbital analysis, as well as electron affinity calculations, were carried out, in a similar way to that performed on α,β-unsaturated analogues [26].

In Table S2 (Supplementary Materials) the main geometrical parameters, at the B3LYP/6-31G(d) level, for the anion radicals from all molecules are presented. The most significant variations, with regard to the neutral species, were the elongation of C7=C8 and C9=O9 double bonds.

**Figure 3:**
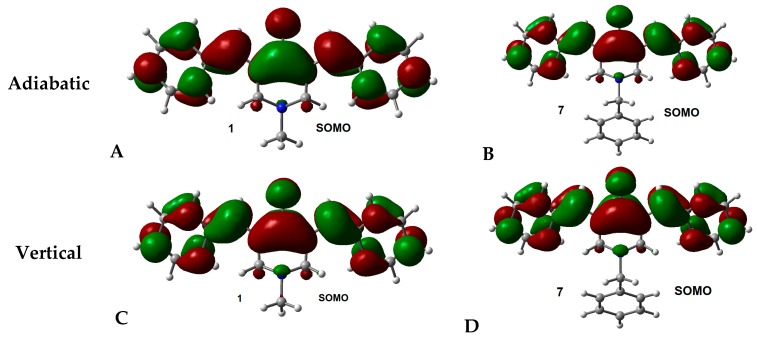
SOMO of adiabatic and vertical anions for molecules **1** and **7**. Isosurface value = 0.02 e/Å^3^.

To study the distribution of the excess electron density in the anion radical of all molecules, the single occupied molecular orbital (SOMO) of the adiabatic and vertical cases were obtained. In dipole-bound anions, the SOMO is distributed in a limited part of the molecule, while in valence-bound anions the SOMO is distributed over the whole molecule. In Figure 3, the SOMO of adiabatic and vertical anions for molecules **1** (representative example for series **1**–**6**) and **7** (representative example for series **7**–**12**), are displayed (others are presented in Figure S3, Supplementary Materials). The representations of SOMOs show that all molecules display a delocalized distribution of the orbital and, therefore, suggest a valence-bound anion in both cases, vertical and adiabatic. LUMOs for vertical and adiabatic anions present the same distribution for **1** to **6** (Figure S3, Supplementary Materials), but are different for **7**–**12** (Figure S4, Supplementary Materials).

Calculated AEAs, VEAs, and VDEs at B3LYP/6-31G(d) and B3LYP/6-311+G(2df,p)// B3LYP/6-31G(d) are presented in Table 3. In order to ensure the adequacy of our approach, AEAs for compounds **1** and **7** were also obtained from geometries optimized at the B3LYP/6-311+G(2df,p) level, and the results (1.398 eV and 1.407 eV respectively) were very close to those obtained at the B3LYP/6-311+G(2df,p)//B3LYP/6-31G(d) level. This shows that high level optimization is not necessary to obtain reliable results. Positive VEA and AEA indicates the favorable tendency of the molecule to attach an electron, the first represents the generation of radical anion without the geometrical rearrangement, while the second indicates that the radical anion is in their final optimized geometry. A positive VDE indicates that the energy of the neutral molecule is higher than their anion, and it is stable with respect to the vertical electron auto-detachment. VEA and VDE can be interpreted as lower and upper bounds of AEA, as long as the nuclear configuration of the anion and the neutral molecule are similar. AEAs for all molecules were positive, which showed that all have a favorable tendency to form stable anion radicals. Positive VEAs for all molecules also indicate that they have a favorable tendency to attach an extra electron. Positive VDEs for all studied molecules indicate that the electron auto-detachment from the anion radical is unlikely to occur.

To study the effect of substituents at the aromatic rings regarding the attachment and detachment of an electron, we correlated the AEAs, VEAs, and VDEs with the Hammett sigma constant [27,28,29]. In both series, **1**–**6** and **7**–**12**, we found for AEA a correlation coefficient (R^2^) of 0.89 (**1**–**6**) and 0.90 (**7**–**12**) for values calculated with the 6-31G(d) basis set and 0.94 (**1**–**6**) and 0.91 (**7**–**12**) for values calculated with the B3LYP/6-311+G(2df,p) basis set (Figures S5 and S6, Supplementary Materials), which indicates a better fit for values calculated with the larger basis. Similarly for VEAs, we found a correlation coefficient (R^2^) of 0.90 (**1**–**6** and **7**–**12**) for values calculated with the 6-31G(d) basis set and 0.91 (**1**–**6** and **7**–**12**) for values calculated with the B3LYP/6-311+G(2df,p) basis set. These results indicate that substituents on the aromatic ring have a significant role on the capability of these molecules to attach an additional electron. VDEs values obtained with the 6-31G(d) and 6-311+G(2df,p) basis sets present similar correlations. Series **1**–**6** exhibits a R^2^ of 0.81, while series **7**–**12** present a R^2^ of 0.89 with both basis sets. The correlation of AEAs, VEAs, and VDEs with the Hammet parameters show that using an extended basis set, such as B3LYP/6-311+G(2df,p), to calculate electronic energy and properties, do not significantly improve the results. 

Compounds **4** and **10**, with the strongest electro-donor *p*-N(CH_3_)_2_ group, on the aromatic rings, presented the lowest AEAs, followed by the unsubstituted compounds **1** and **7**, and by the *p*-Cl compounds **2** and **8** and the *p*-Br derivatives **3** and **9**, which can be considered weak electron-donor substituents at long distances. Compounds with strong electron-acceptor substituents as –CF_3_ (**6** and **12**) and –CN (**5** and **11**) on *para* position of the aromatic rings had higher AEAs. The relationship between substituents on the aromatic rings with VEAs and VDEs was the same that for AEAs. These results also agree with basic organic chemistry principles. 

**Figure 4:**
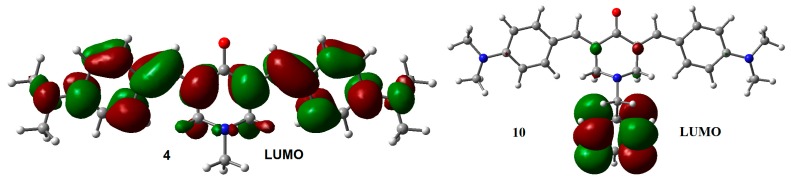
LUMO for adiabatic radical anions **4** and **10.** Isosurface value = 0.02 e/Å^3^.

A particularly interesting issue is the difference observed in the VDE values between compounds **4** and **10**. The slightly larger difference in the VDE between **4** and **10**, compared with the difference between the other coupled analogs, suggests that the excess electron density arising from the *p*-N(CH_3_)_2_ substituents on the lateral aromatic rings, should increases the probability to populate excited states, allowing electron transfer between the central aromatic ring and the rest of the structure. The latter can explain the difference observed in the VDE between **10** and **4**. LUMO distribution in **4** and **10** (Figure 4) support this explanation. 

### 2.3. Local Reactivity

To study the effect of substituents on the reactivity towards nucleophiles, Fukui function (*f*^+^), and Parr function (*P*^+^) were calculated [30,31]. We focused on the atoms that can experience a nucleophilic attack, atoms C_7_ and C_9_ at the α,β-unsaturated ketone C_7_=C_8_-C_9_=O_9_ fragment. It has been previously demonstrated that the addition of biological nucleophiles to this structures plays a key role in the anticancer activity of these curcumin analogues [32], with selective reactivity towards the thiol group, a soft nucleophile [16]. Nucleophile-electrophile reactions occur following the hard and soft acid and base (HSAB) theory, which indicates that nucleophiles react preferentially with electrophiles of similar hardness or softness [33]. The oxygen carbonyl atom O_9_ withdraw electron density from C_7_=C_8_, which creates a deficiency of electron density at C_7_. The later gives as a result a relatively hard carbonyl carbon atom (C_9_) for a 1,2-addition, and a relatively soft β-carbon atom (C_7_) for a 1,4-addition. 

Fukui function (*f*^+^) and Parr function (*P*^+^) for nucleophilic attack toward C_7_ and C_9_ are presented in Table 4. According to *f*^+^, C_7_ is slightly more reactive than C_9_ toward nucleophiles for all molecules. *P*^+^ indicate reactivity in the same direction, but a more significant difference in the electrophilic reactivity for C_7_ with regard to C_9_ is predicted.

According to both reactivity indices, analogues with N(CH_3_)_2_ substituents in the aromatic rings (**4** and **10**) present the greatest difference in reactivity between C_7_ and C_9_, while analogues with CN substituents in the aromatic rings (**5** and **11**) present the lowest difference. 

## 3. Materials and Methods

The calculations were carried out using the Gaussian09 program package [34], running in a Supermicro cluster of blades. In order to find the minimum energy conformation, compound **7** geometry was optimized at the DFT B3LYP/6-31G(d) level and, later, a semi-empirical PM6 level conformational analysis was carried out on the rotations around N_11_-C_12_ and C_12_-C_Ar_ bonds. After that, compound **7** was re-optimized at DFT B3LYP/6-31G(d) starting from the minimum energy conformation found at PM6 level. This minimum energy conformation found for **7** was used as the initial model for the optimization of **8**–**12**. No symmetry constraints were imposed to the optimization of the other molecules, which were performed at DFT B3LYP/6-31G(d) level. No imaginary vibrational frequencies were found at the optimized geometries, indicating that they are true minima of the potential energy surface.

Electron affinities and vertical detachment energies were calculated as follow:

Adiabatic electron affinity (AEA) = *E*(optimized neutral) − *E*(optimized anion) (1)

Vertical electron affinity (VEA) = *E*(optimized neutral) − *E*(anion at optimized neutral geometry) (2)

Vertical detachment energy (VDE) = *E*(neutral at optimized anion geometry) − *E*(optimized anion) (3)

where *E* = *E*_elec_ + zero-point vibrational energies (ZPVE).

Gas phase energies were also obtained at the B3LYP/6-311+G(2df,p) level, through single point calculations based on B3LYP/6-31G(d) optimized geometries. ZPVE obtained at B3LYP/6-31G(d) were used as correction for B3LYP/6-311+G(2df,p) energies. This methodology has shown good results in similar calculations [35]. In order to ensure the adequacy of our approach, AEAs for compounds **1** and **7** were also obtained from geometries optimized at the B3LYP/6-311+G(2df,p) level. 

Global reactivity indices, such as electronic chemical potential (μ), hardness (η), and electrophilicity (ώ), were obtained for all molecules according with their definitions [20,21]. Local indices, Fukui function (*f*^+^) and Parr function (*P*^+^), for electrophilic attack were calculated as previously described [31,31].

## 4. Conclusions

In this work, we have studied the molecular structure of twelve *N*-substitued-4-piperidone curcumin analogues, focusing on the effect of substituents on geometries, frontier molecular orbitals, electron affinities, and global, as well as local, reactivity indices. Methyl and benzyl substituents on the nitrogen atom do not have significant effects on the geometries and frontier molecular orbitals of both neutral and radical anion species, showing that the proper choice of groups located at the nitrogen atom can be a nice procedure to modify relevant properties linked to biological activities, such as lipophilicity, solubility, etc., without affecting the reactivity of the molecule. On the other hand, substituents on the aromatic rings influence the distribution of HOMO orbitals of the neutral molecules, but do not affect the SOMO of radical anions. Additionally, the LUMO do not seems to be affected by the substituents, not in neutral form, neither as a radical anionic species. Except for compound **10** where the LUMO orbital displays a significant population at the methylene group. 

Adiabatic and vertical electron affinities (AEA and VEA), as well as vertical detachment energy (VDE), were calculated at the B3LYP/6-31G(d) and B3LYP/6-311+G(2df,p) levels. The effect of substituents on the aromatic rings was studied by correlations with the Hammett sigma constant. As expected, for AEAs the correlation coefficients present a better fit with the 6-311+G(2df,p) than with the 6-31G(d) basis set. For VEAs and VDEs no differences were found. The analysis of the SOMO orbital distribution for both adiabatic and vertical radical anions, as well as the difference between AEA and VEA, indicates that for all studied molecules, the formed anion radicals are valence-bound types.

The Fukui (*f*^+^) and Parr functions (*P*^+^) for electrophilic attack were calculated and show the influence of the substituent on the aromatic rings on the reactivity of α,β-unsaturated ketone systems toward nucleophiles. C_7_ showed to be more reactive toward nucleophilic attack than C_9_, which indicates that 1,4-addition is more favorable than 1,2-addition. Furthermore, the difference in local reactivity on these electrophilic sites was influenced by the nature of the substituent present in the aromatic rings. The results found in this work can be useful for the design of curcumin analogues based on a 4-piperidone core with desired reactivity.

## Figures and Tables

**Table 1:**
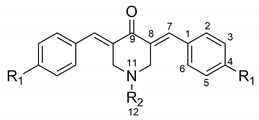
Molecules studied in this work.

Compounds	R_1_	R_2_
**1**	-H	CH_3_
**2**	4-Cl	CH_3_
**3**	4-Br	CH_3_
**4**	4-NMe_2_	CH_3_
**5**	4-CN	CH_3_
**6**	4-CF_3_	CH_3_
**7**	-H	CH_2_Ph
**8**	4-Cl	CH_2_Ph
**9**	4-Br	CH_2_Ph
**10**	4-NMe_2_	CH_2_Ph
**11**	4-CN	CH_2_Ph
**12**	4-CF_3_	CH_2_Ph

**Table 2:** HOMO, LUMO, and GAP HOMO-LUMO (GAP_H-L_) energy, electronic chemical potential (μ), hardness (η) and electrophilicity (ώ) ^a^.

Compounds	HOMO	LUMO	GAP_H-L_	μ	η	ώ
**1**	−6.01	−2.09	3.92	−4.05	1.96	4.18
**2**	−6.22	−2.37	3.85	−4.30	1.92	4.81
**3**	−6.21	−2.38	3.83	−4.30	1.91	4.81
**4**	−4.87	−1.51	3.36	−3.19	1.68	3.03
**5**	−6.54	−2.92	3.62	−4.73	1.81	6.18
**6**	−6.36	−2.59	3.84	−4.48	1.89	5.31
**7**	−5.91	−2.06	3.85	−3.99	1.92	4.14
**8**	−6.11	−2.34	3.77	−4.22	1.88	4.74
**9**	−6.11	−2.34	3.77	−4.22	1.88	4.74
**10**	−4.87	−1.50	3.37	−3.18	1.68	3.01
**11**	−6.41	−2.88	3.53	−4.65	1.77	6.11
**12**	−6.22	−2.55	3.67	−4.38	1.84	5.21

^a^ All values in eV.

**Table 3:** Calculated Adiabatic Electron affinities (AEAs), vertical electron affinities (VEAs), and vertical detachment energies (VDEs) for molecules **1**–**12** in vacuum with the B3LYP/6-31G(d) ^a^, and B3LYP/6-311+G(2df,p)// B3LYP/6-31G(d) ^b^, basis sets ^c^.

Compounds	AEA		VEA		VDE	
	Basis 1 ^a^	Basis 2 ^b^	Basis 1 ^a^	Basis 2 ^b^	Basis 1 ^a^	Basis 2 ^b^
**1**	0.932	1.398	0.791	1.295	1.059	1.556
**2**	1.263	1.669	1.124	1.571	1.391	1.829
**3**	1.280	1.707	1.144	1.606	1.406	1.859
**4**	0.578	0.977	0.361	0.839	0.851	1.319
**5**	1.856	1.902	1.718	2.181	1.998	2.454
**6**	1.512	2.002	1.332	1.856	1.685	2.202
**7**	0.939	1.407	0.798	1.303	1.062	1.560
**8**	1.264	1.672	1.122	1.571	1.386	1.826
**9**	1.282	1.709	1.142	1.606	1.400	1.855
**10**	0.531	0.970	0.379	0.858	0.690	1.167
**11**	1.848	2.271	1.706	2.170	1.985	2.443
**12**	1.510	1.999	1.324	1.849	1.676	2.193

^c^ AEA, VEA, and VDE in eV.

**Table 4:** Fukui function (*f*^+^) and Parr function (*P*^+^) for nucleophilic attack at C_7_ and C_9_.

Molecule	Fukui Function for Nucleophilic Attack		Parr Function for Nucleophilic Attack	
	C_7_	C_9_	C_7_	C_9_
**1**	0.062	0.057	0.199	0.121
**2**	0.059	0.054	0.185	0.113
**3**	0.059	0.054	0.182	0.111
**4**	0.063	0.054	0.205	0.124
**5**	0.049	0.047	0.129	0.082
**6**	0.056	0.053	0.160	0.099
**7**	0.061	0.056	0.198	0.121
**8**	0.058	0.054	0.183	0.113
**9**	0.058	0.053	0.180	0.112
**10**	0.061	0.053	0.213	0.130
**11**	0.047	0.046	0.127	0.083
**12**	0.055	0.053	0.158	0.099

## Supplementary Materials

Supplementary materials can be accessed at: http://www.mdpi.com/1420-3049/21/12/1658/s1.
