# Supplementary material for: A Computational Study of Structure and Reactivity of N-Substitued-4-Piperidones Curcumin Analogues and Their Radical Anions
Source: Molecules. 2016 Dec 2;21(12):1658. doi: 10.3390/molecules21121658 (PMC6273823; doi:10.3390/molecules21121658)
Supplement: Supplementary file 1 [file molecules-21-01658-s001.pdf]

Supplementary Materials: A Computational Study of Structure and Reactivity of *N*-Substitued-4-Piperidones Curcumin Derivatives and Their Radical Anions

Maximiliano Martínez-Cifuentes, Boris Weiss-López and Ramiro Araya-Maturana

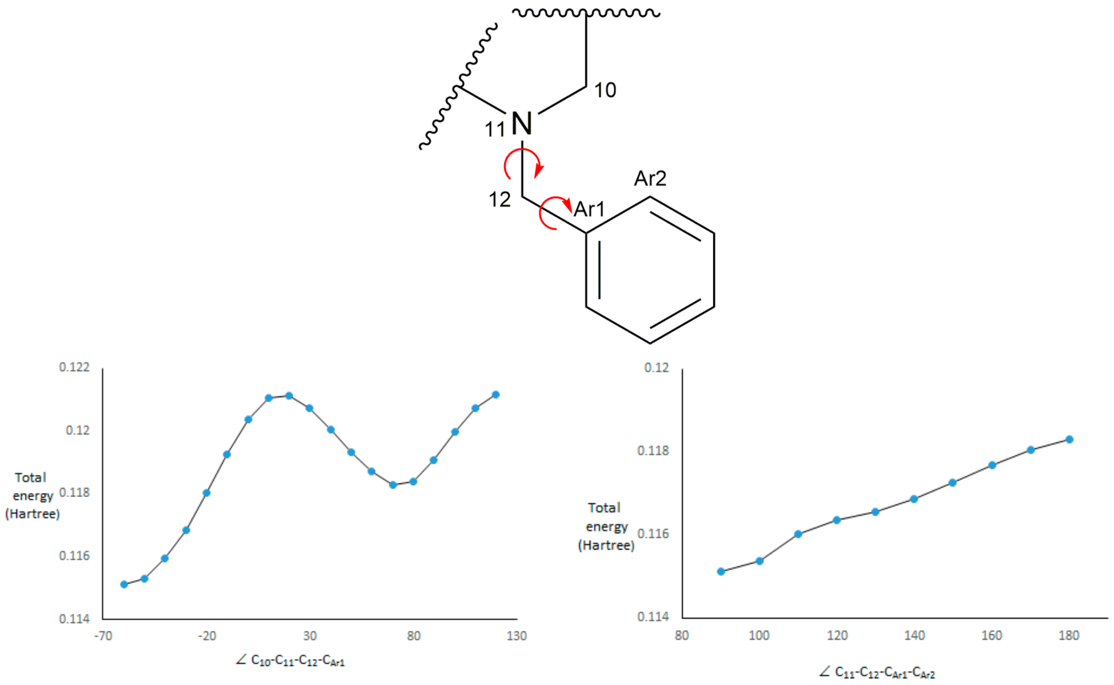

Figure S1. Energy profile for rotation around N_{11}–C_{12} and C_{12}–CAr_1 bonds of compound **7**.

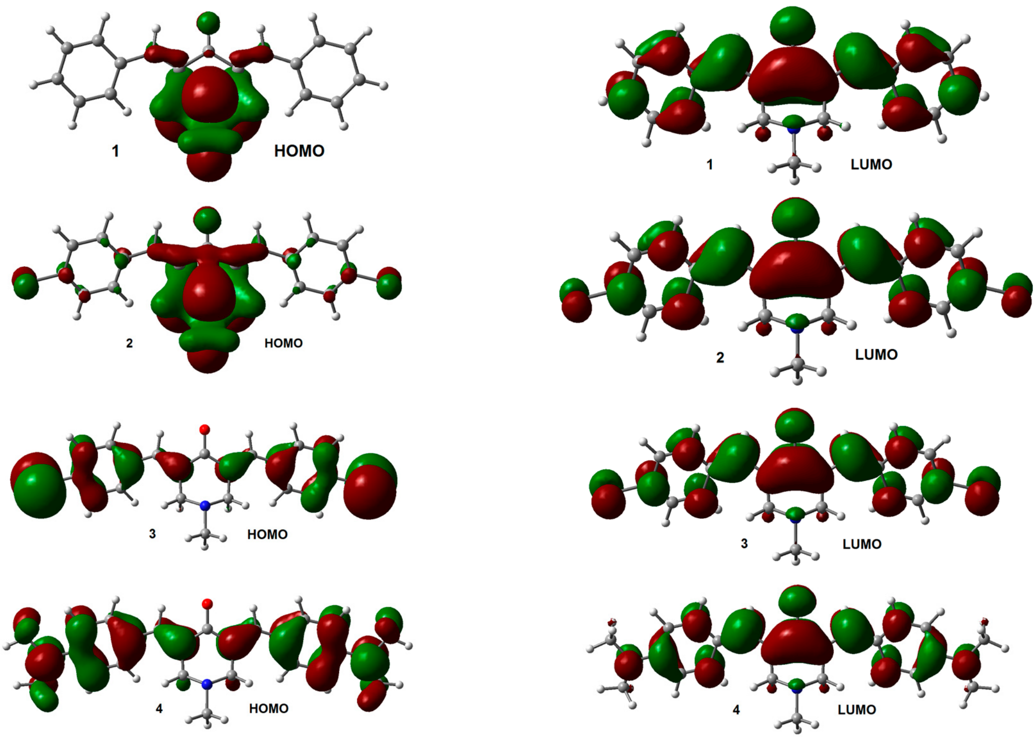

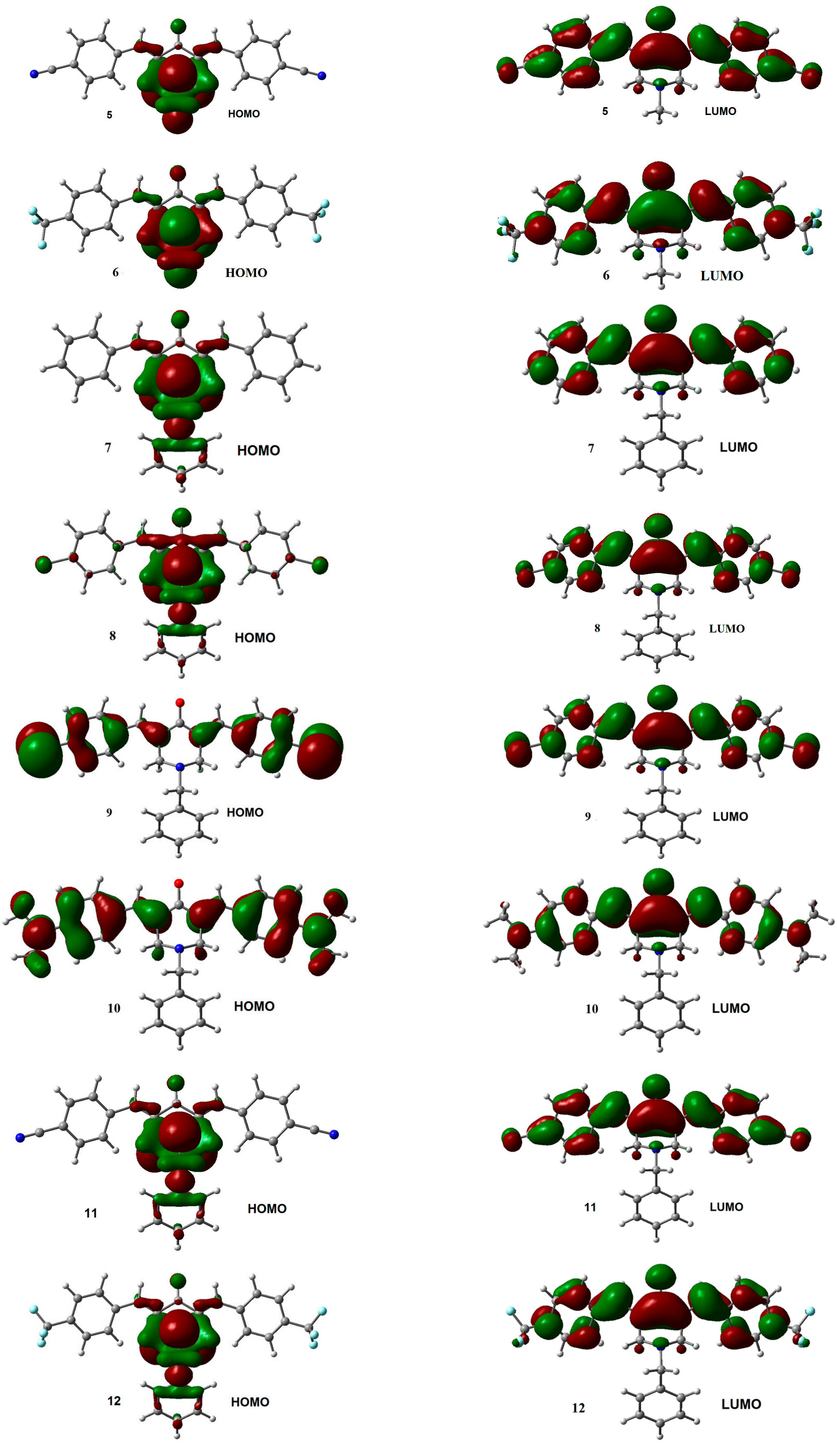

Figure S2. Frontier Molecular Orbitals (FMO) for neutral molecules. Isosurface value = 0.02 e/Å^3

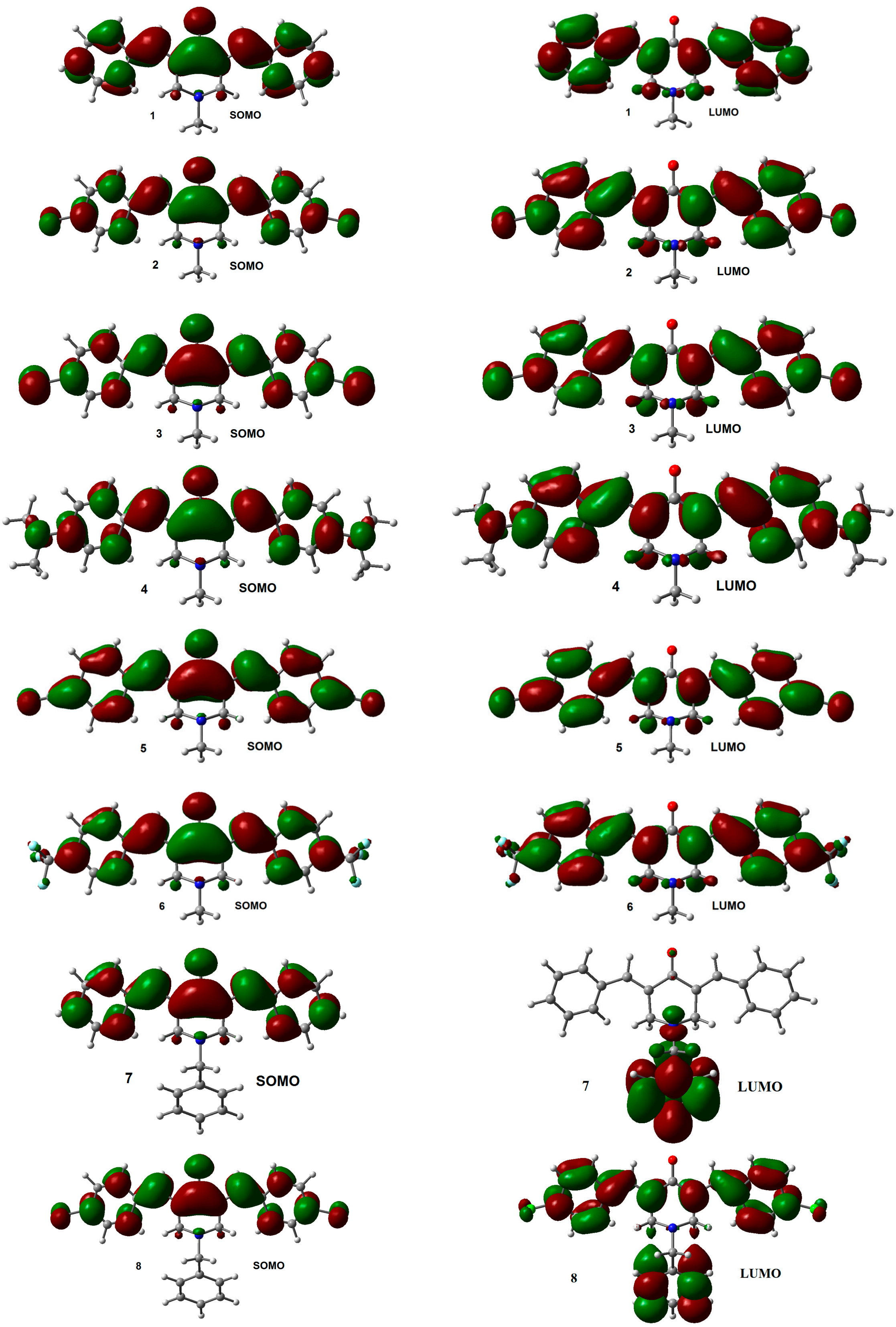
1 SOMO
1 LUMO
2 SOMO
2 LUMO
3 SOMO
3 LUMO
4 SOMO
4 LUMO
5 SOMO
5 LUMO
6 SOMO
6 LUMO
7 SOMO
7 LUMO
8 SOMO
8 LUMO

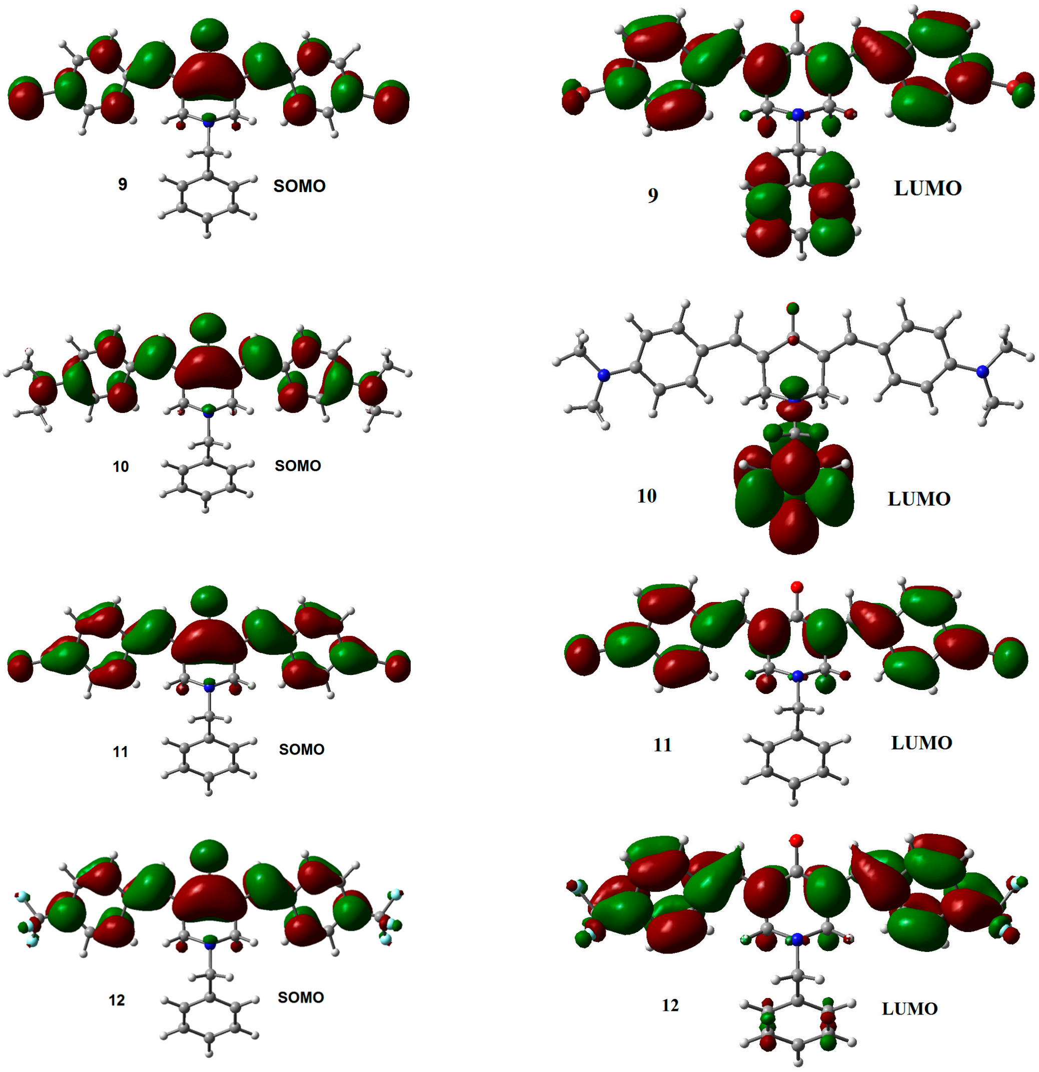

Figure S3. Frontier Molecular Orbitals (FMO) for vertical radical anions (equals to adiabatic, except LUMO for **7**–**12**). Isosurface value = 0.02 e/Å^3.

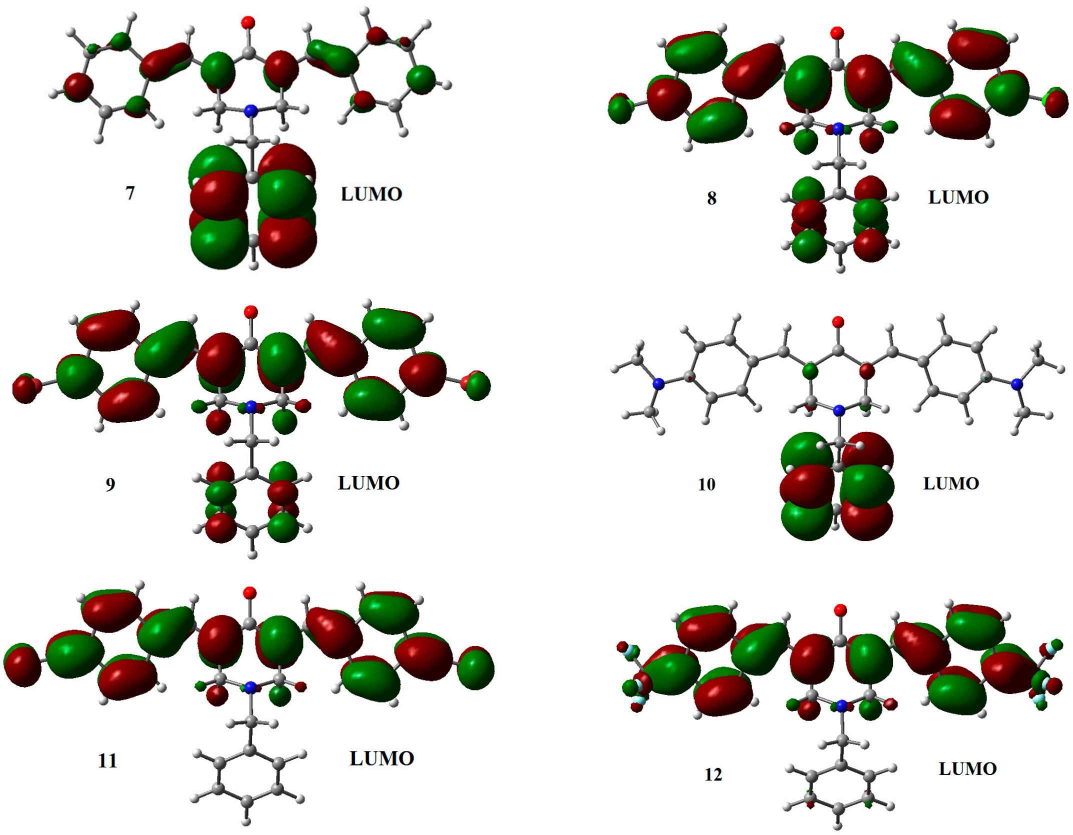

Figure S4. LUMO for adiabatic radical anions **7**–**12**. Isosurface value = 0.02 e/Å^3.

Table S1. Main geometrical parameters for the neutral molecules.

Compounds	C_1–C_2	C_2–C_3	C_3–C_4	C_1–C_7	C_7=C_8	C_9=O_9	N_{11}–C_{12}
**1**	1.410	1.391	1.397	1.463	1.353	1.230	1.455
**2**	1.410	1.390	1.395	1.462	1.353	1.230	1.455
**3**	1.410	1.390	1.395	1.462	1.353	1.230	1.455
**4**	1.411	1.385	1.416	1.456	1.356	1.234	1.453
**5**	1.411	1.387	1.406	1.463	1.353	1.228	1.456
**6**	1.410	1.389	1.398	1.464	1.352	1.229	1.456
**7**	1.410	1.391	1.397	1.464	1.353	1.230	1.470
**8**	1.410	1.390	1.395	1.463	1.353	1.230	1.470
**9**	1.410	1.390	1.395	1.463	1.353	1.230	1.471
**10**	1.411	1.385	1.416	1.456	1.356	1.233	1.468
**11**	1.411	1.387	1.406	1.463	1.353	1.228	1.472
**12**	1.410	1.390	1.398	1.464	1.352	1.229	1.471

Table S2. Main geometrical parameters for radical anions of **1–12**.

Compounds	C_1–C_2	C_2–C_3	C_3–C_4	C_1–C_7	C_7=C_8	C_9=O_9	N_{11}–C_{12}
**1**	1.424	1.388	1.402	1.445	1.377	1.262	1.448
**2**	1.424	1.387	1.398	1.443	1.377	1.261	1.449
**3**	1.424	1.387	1.397	1.443	1.377	1.261	1.449
**4**	1.424	1.383	1.410	1.444	1.377	1.263	1.448
**5**	1.426	1.380	1.415	1.439	1.376	1.256	1.450
**6**	1.425	1.383	1.406	1.441	1.376	1.259	1.449
**7**	1.423	1.388	1.402	1.445	1.377	1.262	1.461
**8**	1.423	1.387	1.398	1.443	1.377	1.261	1.462
**9**	1.424	1.387	1.397	1.443	1.376	1.261	1.462
**10**	1.420	1.387	1.413	1.445	1.377	1.264	1.461
**11**	1.426	1.380	1.415	1.439	1.375	1.256	1.464
**12**	1.410	1.390	1.398	1.464	1.352	1.229	1.471

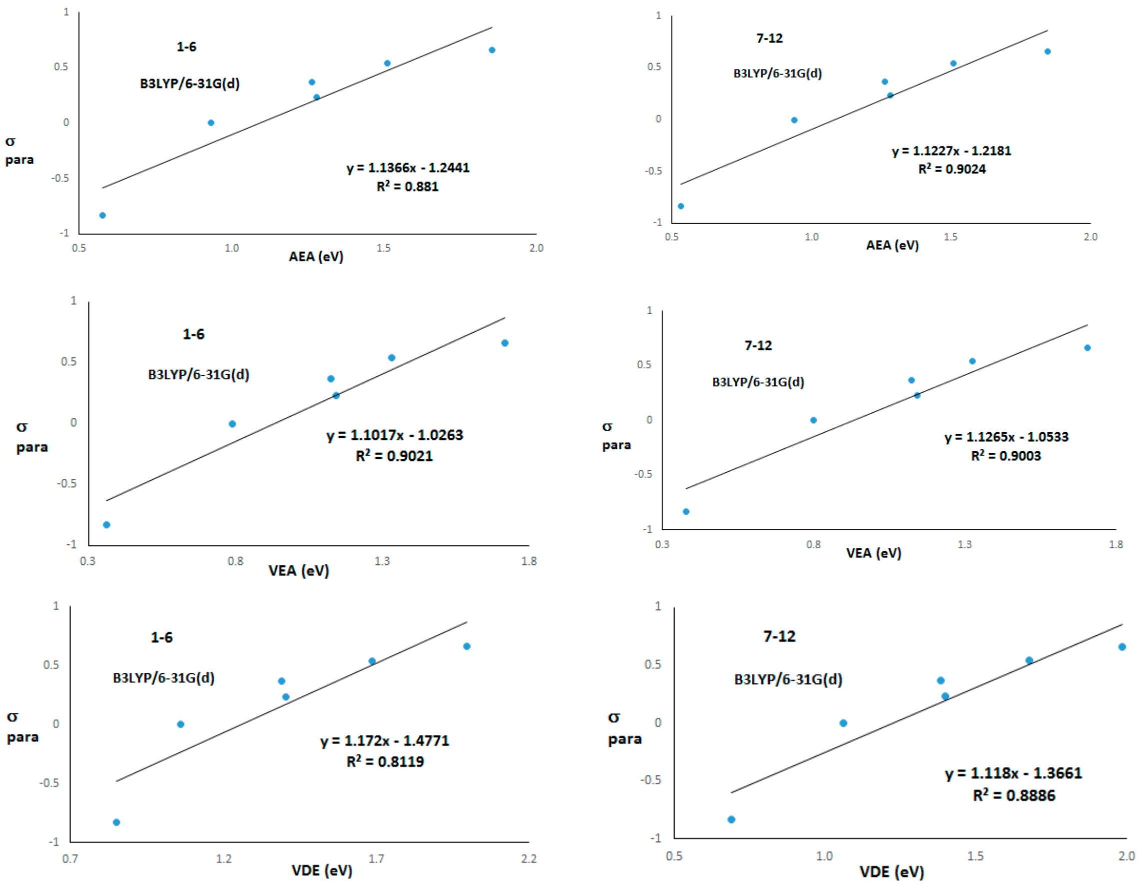

Figure S5. Correlation among AEAs, VEAs and VDEs with Hammett sigma constant at B3LYP/6-31G(d).

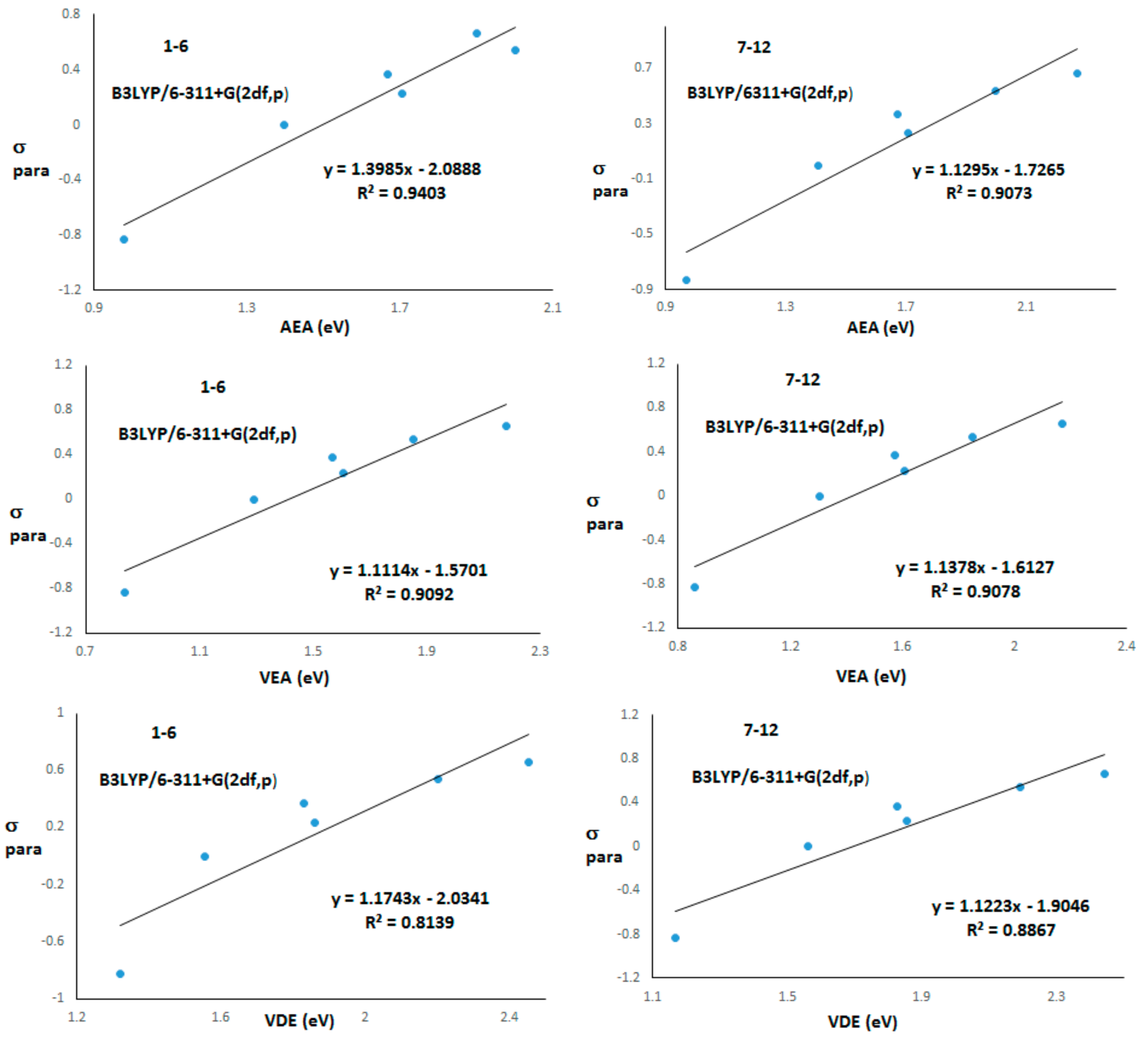

Figure S6. Correlation among AEAs, VEAs and VDEs with Hammett sigma constant at B3LYP/6-311+G(2df,p)//B3LYP/6-31G(d).
